# Möbius Band Surprise: A Systematic Illusion in Imagery

**DOI:** 10.1177/20416695211004972

**Published:** 2021-04-09

**Authors:** Mons Daniel Haugland, David G. Pearson, Vebjørn Ekroll

**Affiliations:** Department of Psychosocial Science, 1658 University of Bergen, Norway; School of Psychology and Sports Science, Anglia Ruskin University, United Kingdom; Department of Psychosocial Science, 1658 University of Bergen, Norway

**Keywords:** Möbius band, magic trick, visual imagery, spatial reasoning, connectedness, attribute substitution, cognitive illusion, cognitive failure

## Abstract

When a Möbius loop is cut along the middle of the band, the result is a single connected loop, yet anecdotal evidence from science demonstrations and the use of this effect in magic tricks suggest that most people are thoroughly surprised by this because they strongly believe that the result should be two separate loops. Here, we present results from a behavioral experiment confirming this anecdotal evidence and discuss potential theoretical explanations for why this demonstration evokes strong, but misleading intuitions and a related illusion of impossibility.

The well-known Möbius band (Figure 1B), which can be obtained by joining the two ends of a paper strip (Figure 1A) to a loop after twisting one of the ends by 180 degrees, has many curious and counterintuitive properties. One of these is that if you cut the band along the middle all the way around the loop, you end up with a single unbroken loop rather than two separate loops (Gardner, 1977; Tokieda, 2014). Anecdotal evidence suggests that what happens here appears impossible or even magical to most people, and the effect is indeed exploited in a magic routine known as The Afghan Bands (Gardner, 1977; Wilson, 1988). That people experience something that is very well possible as impossible (Kuhn, 2019; Macknik, King et al., 2008; Macknik, Martinez-Conde et al., 2010) is of course a phenomenon that warrants explanation, and in this regard, the Möbius strip is not only of mathematical interest but also poses a challenge to cognitive science (Ekroll, 2019).

**Figure 1.**
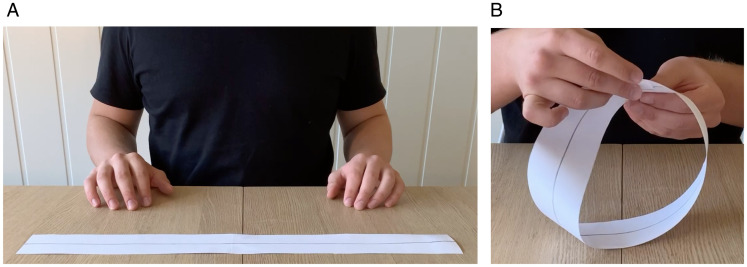
When the ends of a paper strip (A) are glued together after one of the ends has been twisted by half a turn (180 degrees), a Möbius band (B) is obtained. Cutting the Möbius along the middle (gray line) all around the loop, yields a counter-intuitive result, namely a single unbroken loop, rather than two separate loops.

The purpose of the present experiment was to establish to what extent people actually have difficulties in reasoning about the outcome of cutting the Möbius band, and to what extent their expectations are shaped by a heuristic biasing them to expect two objects, as the anecdotal evidence suggests. We asked our participants to predict the results of cutting a standard Möbius band (Supplementary Movies 1 and 3), as well as a slightly more complicated modification of it (Supplementary Movies 2 and 4), where the strip is twisted by 360 degrees (rather than 180 degrees) before it is joined into a loop (Gardner, 1977; Tokieda, 2014). While cutting the standard Möbius band results in a single loop (Supplementary Movie 5), cutting the modified Möbius results in two loops (which are interlinked, see Supplementary Movie 6).

To anticipate, the results suggest that people perform very poorly in reasoning about the result of cutting the Möbius strip, and that they almost always instead rely on a heuristic biasing them to expect two parts or abstract knowledge based on familiarity with this type of effect. After presentation of the experimental findings, we discuss potential explanations for the difficulty in reasoning about the Möbius strip and the tendency to expect two parts.

## Experiment

### Methods

The participants in our study viewed two different pairs **A** and **B** of movies presented on Powerpoint slides in a lecture hall setting. One pair (Supplementary Movies 1 and 3) showed the standard Möbius loop (where the band is twisted by a *half turn*) and the other pair (Supplementary Movies 2 and 4) showed a modified version (where the band is twisted by a *whole turn*). The first movie in each pair showed how the experimenter constructed the loop in question by gluing together the two ends of a paper strip, while the second movie just showed the finished loop being moved about and rotated a little in order to provide a good spatial impression of it. The paper strips used to construct the loops had a clearly visible black line drawn along the middle to illustrate how the loops were supposed to be cut. The construction of the loops in the former movies was accompanied by audio tracks of the experimenter carefully explaining each step in the construction. The audio tracks in Supplementary Movies 1 and 3 are in English, but the audio tracks actually used in the experiment were in Norwegian (Supplementary Movies 7 and 8). The English transcripts are also provided in the Appendix. At the end of the construction movies, when the loops were finished, the experimenter asked “What will you end up with, if you cut along the line in the middle the whole way around?”. After that, a new Powerpoint slide was shown, where the question was repeated in writing and the second movie illustrating the same loop was running in loop mode while the participants filled in a response sheet containing two written specifications of the same question, namely, “Describe what you will end up with if you cut along the line in the middle of the object:” and “Draw a simple sketch to illustrate your description:”. The participants were asked to complete their responses within 4 minutes. After that, they were asked to turn their response forms around. The backside contained the same two questions, and the procedure was repeated for the second set of movies showing the other version of the Möbius loop. The two sides of the response sheet were identical apart from the heading which read “Task A” at the first page and “Task B” at the second page. After completion of the second round, a new slide was shown with the question “How many pieces do you think would have resulted in Task A? State the number in the top right corner at the page for Task A.” Afterwards, a new slide was shown with the same question for Task B.

The main reason why we asked the participants to provide descriptions and drawings of what they thought would result before asking explicitly about how many of pieces they thought would result was that we suspected that the explicit question about numbers may influence their response. If it appears self-evident to a participant that two pieces would result, asking explicitly about the number of pieces may nudge him or her towards questioning this assumption and consider other alternatives.

The experiment was performed with two groups of university students in the break between two 45-minute parts of a first-year introductory lecture in social psychology. Both groups of students attended the same lecture in terms of content but were enrolled in different programs which attended the lecture at different times. In one group, the half twist Möbius band was presented first, and the whole twist modification second. In the other group, the presentation order was reversed.

Prior to the experiment, the participants were informed that the aim of the study was to investigate “apprehension of spatial objects” and provided written informed consent. To encourage participation, one participant in each group was randomly drawn to win 500 NOK (about 45€) in cash and two participants in each group were randomly drawn to each win two cinema vouchers.

### Results

In total 261 participants returned a response sheet, 146 participants in the group where the half-twist stimulus was presented first and 115 in the group where the whole-twist stimulus was presented first.

For each participant and condition, author M. H. evaluated how many pieces the participant believed would result after cutting the loops along the middle, by considering the combined evidence from the participant’s verbal description and drawing. He also evaluated two further properties of the objects depicted in the participants’ drawings that are informative about the correctness of the response. First, he judged whether the strip(s) were depicted as closed or open. In the half turn condition, a single closed strip is correct, while an open strip (i.e., a strip with two free ends that are not joined together) is wrong. The same holds for the whole turn condition, except that there are two closed strips. Second, whenever participants (correctly) drew two loops in the whole turn condition, he also evaluated whether the two loops were interlinked (correct) or not (wrong). Based on these separately evaluated aspects (number of pieces, closedness, and interlinkedness) of the responses, a given single response was coded as *completely correct* whenever they were correct with respect to all of them.

Cases where the participant had not filled in the form for the particular task, or the description and drawing were impossible to interpret were treated as missing values. More specifically, data obtained by evaluating the verbal descriptions and drawings of a given participant were only analyzed if it was codable with respect to all the three above criteria for both stimuli. This left us with 219 valid response pairs. Similarly, data resulting from the participants’ explicit statements regarding the resulting number of pieces were only analyzed if they were provided for both stimuli. This left us with 248 valid response pairs.

**Figure 2.**
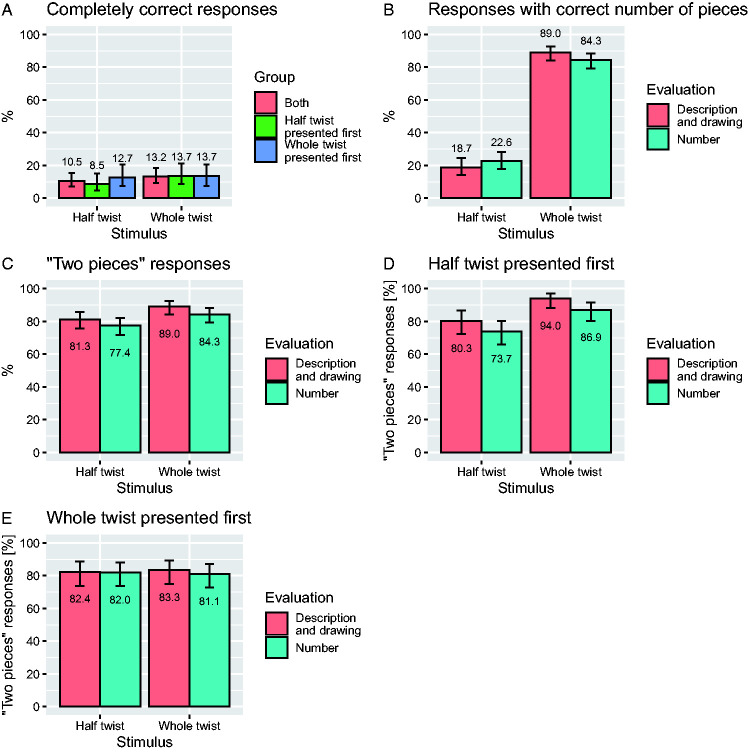
A: Percentage of completely correct responses for the standard Möbius loop (half twist) and the modified version (whole twist). B: Percentage of responses indicating the correct number of pieces. The magenta bars show the results based on evaluation of the participants’ descriptions and drawings and the cyan bars show the results based on the numbers stated explicitly by the participants. Note that the correct response is “1 piece” for the half-twist stimulus and “2 pieces” for the whole-twist stimulus, hence a wrong response for the half-twist stimulus is a “2 piece” response. C: Same data plotted in terms of the percentage of “2 pieces” responses. D: Same as (C), but plotted separately for the condition where the half twist loop was presented first. E: Same as (C), but plotted separately for the condition where the whole twist loop was presented first. The error bars show 95% binomial proportion confidence intervals.

Figure 2A shows the percentages of completely correct responses for the two stimuli, as evaluated based on the participants’ description and drawings. These percentages are shown for the two groups (who viewed the two stimuli in different order) combined, as well as separately for each group. As can be seen, the percentages are all quite low. The slightly higher overall percentage for the whole-twist stimulus (13.2%) was not significantly different (*p* = .36, odds ratio 1.5, McNemar’s test) from the percentage for the half-twist stimulus (10.5%). The small differences between the two groups were also not statistically significant (*p* = .38, odds ratio 1.56 for the half-twist stimulus and *p* = 1, odds ratio 0.92 for the whole-test stimulus, both Fisher’s exact tests).

Figure 2B shows the percentages of responses that were correct with respect to the resulting number of pieces (but not necessarily with respect to the other criteria). Compared to the percentages of completely correct responses (Panel A), these percentages are higher, which is unsurprising, since fulfilment of additional criteria was required for a response to be coded as completely correct, but it is notable that they are *much* higher for the whole-twist stimulus (89% based on the description and drawings and 84.3% based on the explicitly stated numbers).

Remember that the response “2 pieces” is wrong for the half-twist condition, but correct for the whole-twist condition. Panel C replots the percentages in Panel B as percentages of “2 pieces” responses. The tendency to respond “2 pieces” is similar and strong for both stimuli, but somewhat larger for the whole-twist stimulus (89% based on the description and drawings and 84.3% based on the explicitly stated numbers) than for the half-twist stimulus (81.3% and 77.2%, respectively), and the difference is statistically significant (*p* = .010, odds ratio 2.55 and *p* = .034, odds ratio 1.85, respectively, both McNemar’s tests), but the difference is rather small.

Panels D and E show the same as Panel C, but plotted separately for the group of participants to whom the half-twist stimulus was presented first and the group of observers to whom the whole-twist stimulus was presented first. In the former group (Panel D), the difference between the two stimuli (13.7% based on the description and drawings and 13.1% based on the explicitly stated numbers) is more pronounced and also statistically significant (*p* = .001 and *p* = .006, respectively). In the latter case (Panel E), this difference is small (approximately 1% in both cases), goes in the opposite direction in the case of the explicitly stated numbers, and is not statistically significant (*p* = 1 in both cases). The larger effect of stimulus type in the group of participants to whom the half-twist stimulus was presented first (Panel D) than in the group of participants to whom the whole-twist stimulus was presented first (Panel E) was also statistically significant (*p* = .025 based on the description and drawings and *p* = .018 based on the explicitly stated numbers, both Wilcoxon tests).

## Discussion

The majority (about 80%) of the participants in our study erroneously predicted that cutting the standard Möbius band along the middle will produce two separate pieces, although this operation actually produces a single unbroken loop. This finding confirms the anecdotal evidence from magic tricks and science demonstrations suggesting that peoples’ intuitions about what will happen here are systematically misleading. The specific source of this misleading intuition is presently unknown, but Ekroll (2019) has suggested that it may be due to some form of attribute substitution (Kahneman & Frederick, 2002). The general notion behind attribute substitution is that “when confronted with a difficult question people often answer an easier one instead, usually without being aware of the substitution” (Kahneman & Frederick, 2002, p. 53). When trying to figure out what will happen when the Möbius strip is cut along the middle, people may unconsciously substitute the Möbius strip with a simple untwisted loop and therefore come to the conclusion that two separate pieces will result. Other specific forms of attribute substitution may also explain the effect. For instance, the path of the cut made by the scissors along the Möbius band may be substituted by a plane intersecting the loop, which would indeed imply two separate pieces (Ekroll, 2019).

Regardless of the specific details of this account, however, the general hypothesis is that the intuition that the cutting will produce two separate pieces is due to automatic heuristic processing (Stanovich & West, 2000). Assuming that automatic system-1-like processes generate this intuition quickly and effortlessly for everybody, a correct response to the half twist Möbius band (“a single connected piece”) will only ensue if participants base their response on some other source of knowledge than their immediate intuition. Two options seem plausible here. First, a participant may arrive at the correct response by engaging in effortful system-2-like processing. Second, she or he may already be familiar with the particular effect and know the correct answer on an abstract level.

Due to the limited time slot available for our data collection, we had to keep our experiment short and therefore did not ask our participants whether they were familiar with the effect from before. Therefore, it is difficult to tell to what extent the relatively few responses to the half-twist stimulus that were correct with respect to the number of resulting pieces (one) are due to system-2-like processing or mere familiarity with the effect from before. Even so, it is clear from our results that successful system-2-like processing of the half-twist stimulus, if it ever occurs at all, is relatively rare since only about 20% provided a response with the correct number of pieces (Figure 2B), and only about 10% provided a response that was correct with respect to all criteria (Figure 2A). On the reasonable assumption that successful system-2-like processing would produce a response that is not only correct with respect to the number of pieces but also with respect to the other criteria, we can conclude that the proportion of participants who engaged in successful system-2-like processing of the half-twist stimulus is at most 10% and probably considerably smaller since this proportion may include (or consist entirely of) participants who were familiar with the effect from before.

Considering that the participants can be expected to provide responses indicating the correct number of pieces (two) for the whole-twist stimulus regardless of whether they rely on system-1-like heuristic processing or system-2-like processing, it is hardly surprising that the large majority of our participants (more than 80%, Figure 2B) provided responses indicating the correct number of pieces (two) for this stimulus. The observation that the proportion of completely correct responses to this stimulus (about 13%, Figure 2A) is much lower strongly suggests that the large majority of the responses indicating the correct number of pieces for this stimulus are also due to heuristic system-1-like processing. Somewhat surprisingly, though, not all of the participants provided responses indicating the correct number of pieces for this stimulus, which would be expected regardless of whether they engaged in system-1-like or system-2-like processing. For this stimulus, it is difficult to see how a participant may come up with an erroneous response indicating a single piece, unless he or she was familiar with the counterintuitive single-piece result for the half-twist stimulus and misapplied that knowledge to the whole-twist stimulus. Thus, the proportion of responses indicating the wrong number of pieces to the whole-twist stimulus (somewhat more than 10%, Figure 2B) may be regarded as an estimate of the proportion of participants who were familiar with the basic effect from before. Since the proportion of participants indicating the correct number of pieces for the half-twist stimulus is comparable, it appears reasonable to speculate that many or even all of the correct responses to the half-twist stimulus may be due to familiarity, which in turn would suggest that successful system-2-like processing does not occur at all. Indeed, given the short time period available for reflection, it is not implausible that successful system-2-like thinking is essentially impossible.

Since the effect of the order of stimulus presentation was investigated using a between-subjects design, and subjects were not randomly assigned to the two groups, it is conceivable that the small effects of stimulus order reflect differences between people rather than genuine order effects. Considering that the two groups of participants were reasonable large and included people with presumably similar demographics; however, it appears reasonable to attribute the differences to the order of presentation.

The following are the main conclusions suggested by the results of the present study:

1. Reasoning about the result of cutting the Möbius loop (and the modified version with a whole twist) seems to be very difficult.
2. There is a strong tendency to expect that two pieces will result, irrespective of whether that is correct, as it is for the whole twist version, or wrong, as it is for the standard Möbius loop. This tendency probably reflects an immediate intuition based on a cognitive heuristic.
3. Although there are some responses to the standard Möbius strip which indicate the correct number of pieces (one), and many more responses to the modified Möbius strip which indicate the correct number of pieces (two), at best very few and possibly even none of the correct responses are due to successful system-2-like thinking. Rather, these responses may reflect the application of previous abstract knowledge and guessing or, in the case of the whole twist version, reliance on the abovementioned intuitive heuristic, which in that case happens to be correct.

The results of our study can be taken to suggest that a large majority experience a misleading intuition that cutting the Möbius strip will result in two separate pieces. We can also conclude that a large majority fail to think correctly about the result of cutting the Möbius strip, either because the strong and misleading intuition inhibit them from engaging in effortful system-2-like thinking, or because the time period they had available for doing so (4 minutes) was too short. Thus, further research clarifying the specific source of this misleading intuition and the difficulty in reasoning correctly about the Möbius loop seem warranted and theoretically interesting.

Viewed from the theoretical perspective offered by the concept of attribute substitution (Kahneman & Frederick, 2002) the misleading intuition occurs for two reasons. First, the problem people are asked to solve in this situation is difficult. Second, the difficulty unconsciously triggers the replacement of the original problem with a simpler one, which is then solved, i. e. the original problem is “solved” via a heuristic. Thus, the central theoretical questions are (1) why the original problem is so difficult, (2) what simplified version of the original problem people actually deal with, and (3) what general cognitive principle(s) of heuristics determine the structure of that simplified version of the problem.

The gist of a potential answer to the first question would be that reasoning about the result of cutting the Möbius loop is difficult because it involves questions of connectedness, and that our cognitive apparatus for establishing connectedness is limited. Determining connectedness is a computationally expensive problem (Minsky & Papert, 1969), and although the visual system has a curious ability to code topological relationships in many cases (e.g., Chen, 2001), it is also clear that there are many systematic limitations in the visual perception of spatial relationships and topology (Koenderink, 1984; Ullman, 1984) and that determining the topological properties of moderately complicated curves requires effortful and “serial” mental curve tracing (Jolicoeur et al., 1986).

The misleading intuition evoked by the Möbius band can be characterized as an “illusion of imagery” (Ekroll, 2019) that reflects the internal structural of our cognitive system. While research in the domains of perception (e.g., Carbon, 2014) and higher-level cognition (e.g., Tversky, 1986) has focused strongly on systematic illusions to elucidate the inherent structure of our mental machinery and the heuristics employed by the system to fulfill its purposes, investigations and discussions of illusions and their significance are, with some notable exceptions (Hinton, 1979; Margolis, 1998a, 1998b; Pani, 1997; Pearson, 1998; Pylyshyn, 2003; Tversky, 2005), much rarer in the field of visual imagery. There is evidence that reasoning involving dynamic manipulation is particularly difficult to simulate accurately using visual imagery alone (Pearson & Logie, 2015; Pearson et al., 1996). Furthermore, while mental imagery is often cited as playing an important role during creative reasoning, the exact nature of its contribution has remained controversial (Pearson, 2007). We believe that a more systematic effort towards discovering, describing, classifying, and explaining systematic illusions of imagery would be very useful for furthering our understanding of visual imagery and spatial reasoning. As the history of mathematics and the discussions about the role of (spatial) intuitions in the development and teaching of mathematics (e.g., Fischbein, 1987) show, powerful and sometimes very misleading intuitions are ubiquitous in the field of mathematics. Since spatial intuitions are not only a source of error and confusion in mathematics, but also a major source of insight, precise knowledge about when spatial intuitions lead astray, and when they can be trusted would be of great value to teachers of mathematics. Although there is little reason to doubt that people differ considerably in mathematical and spatial reasoning ability, there may be many similarities in the way people with different levels of mathematical education and ability experience the same kind of misleading intuition in many situations. Studying the art of magic is perhaps particularly instructive in this regard, because magicians have spent centuries developing and selecting effects with the aim of creating magical experiences that work for everybody in the audience. Thus, if something is a successful magic trick regularly used by magicians, there is good reason to believe that it exploits misleading intuitions which are common to everybody and reflect aspects of our cognitive machinery that are universal (Macknik, Martinez-Conde et al., 2010). Ekroll (2019) provides a preliminary overview and taxonomy of magic tricks presumably based on such universal aspects of visual imagery and visuospatial reasoning, many of which are intimately related to mathematics, such as the present Möbius band demonstration, but this preliminary work probably only scratches the surface of a large and interesting field of inquiry for cognitive science.

It is worth noting that there are many similarities and interconnections between mathematics and magic (Gardner, 2014). Many magic tricks rest on mathematical principles, and many mathematical problems evoke the kind of dual mental state or illusion of impossibility which is characteristic of magical experiences, where two seemingly unquestionable beliefs contradict each other. Cantor’s comment on his own proof that sets of different dimensions have the same cardinality—“I see but I do not believe”—is just one famous example of this (Fischbein, 1987). One may speculate that such conflicts between seemingly unquestionable intuitions and firm knowledge furnish a common source of the enjoyment evoked by both mathematical problems and magic tricks (Leddington, 2016).

## Appendix: English Translation of the Audio Tracks Accompanying the Movies

Supplementary Movie 1 (Half twist condition): “Here, you see a strip of paper. Along the middle you see a line. I will glue the ends together, but first, I twist one of the ends by *half a turn*. Thus, I end up with this object. What will you end up with, if you cut along the line in the middle the whole way around?”

Supplementary Movie 2 (Whole twist condition): “Here, you see a strip of paper. Along the middle you see a line. I will glue the ends together, but first, I twist one of the ends by a *whole turn. That is, by half a turn plus half a turn.* Thus, I end up with this object. What will you end up with, if you cut along the line in the middle the whole way around?”
